# Electric Cell-substrate Impedance Sensing in Biocompatibility Research

**DOI:** 10.2478/joeb-2021-0019

**Published:** 2021-12-30

**Authors:** Andrzej Kociubiński

**Affiliations:** 1 Department of Electronics and Information Technology, Lublin University of Technology, Lublin, Poland

**Keywords:** Bioimpedance, biocompatibility, cell culture, technology

## Abstract

In this paper, the possibility of using cell culture impedance measurements to assess the biocompatibility of a material in contact with cells was analyzed. For this purpose, the Electric Cell-substrate Impedance Sensing (ECIS) method and a commercial measuring device were used. The test substrates with thin-film electrodes made of various metals were prepared using the magnetron sputtering method. The choice of metals was dictated by their varying degrees of biocompatibility. Cultures of mouse fibroblasts were cultured on the prepared substrates. The experiment showed that the complete cycle of culture from attachment and reproduction to apoptosis occurred. The results obtained indicate that it is possible to use the ECIS method to study the influence of metal on cell culture activity.

## Introduction

Lab-on-a-chip systems are devices designed to miniaturize analytical or bioanalytical techniques. They are usually based on sensors and actuators produced in microelectronics and MEMS technologies. In biomedical and biological applications, some parts of the device must be in contact with a living system without producing an adverse effect. In the case of contact with metallic materials, noble metals, particularly gold or platinum are usually used which provides the devices a high degree of biocompatibility. However, the high price of these metals limits their use in disposable devices. In addition, in many cases it is not necessary to use materials with high biocompatibility because the measurement time is so short that it does not affect the measurement results and at the same time does not pose a risk of adverse reactions. Alternatively, using less expensive materials can reduce the cost of conducting research. However, the possibility of using specific materials requires testing their biocompatibility, i.e. their effect on living organisms [1, 2, 3].

Like most medical and biological studies, the assessment of a material's biocompatibility is first done by in-vitro methods. The classic method of monitoring cell cultures, as well as individual cells, is to observe them through a microscope. More recently, an interesting alternative to microscopy has been the use of impedance measurement, which can replace the time-consuming optical method and also extend its capabilities in many applications. The measured electrical parameters of biological cells are typically used to investigate only cells or the health status of the body. As a non-invasive, label-free method, impedance measurements can automatically provide sensitive and quantitative results. These advantages make impedance measurements a widely used cell assay method, especially for live cell analysis and long-term monitoring of them [4, 5].

At the same time, designing and manufacturing devices for this measurement technique can be relatively simple. Using integrated microelectronic, microelectromechanical systems (MEMS), and microfluidic technologies, the applications of the impedance measurement method can be extended to almost all aspects of biology, including: live cell counting and analysis, cytology, cancer research, drug screening, food testing, and environmental safety monitoring [5].

Techniques of measuring electrical parameters of cells can be divided into two ways: when cells are suspended in a medium or are adhered to a substrate. The most commonly used impedance measurement techniques for cell culture monitoring are [6, 7, 8, 9, 10]:

*Impedance Flow Cytometry (IFC)* – a method of counting and determining the size of various types of cells and particles suspended in the flowing electrolyte, this method is based on the measurement of impedance in the area of the aperture;

*Electric Impedance Spectroscopy (EIS)* – a method based on measuring the response of a tested sample to stimulation with a voltage or current signal in a wide frequency range;

*Electric Cell-substrate Impedance Sensing (ECIS)* – based on non-invasive monitoring of the frequency-dependent electrical impedance of the cell culture that covers the gold electrodes during the experiment.

## Materials and methods

### Principles of ECIS

The Electric Cell-substrate Impedance Sensing (ECIS) method used in this paper is based on non-invasive monitoring of the frequency-dependent electrical impedance of cultured cells covering gold electrodes during the experiment. It is an in vitro method that allows analysis of cell activity based on cell structure, morphology, ability to reproduce, divide or move. The wide measurement capabilities have allowed the development of many technical upgrades of this method depending on different applications [11, 12, 13]. Currently, it is used, among others to determine the invasive nature of cancer cells, substance toxicity, or drug testing.

The cell life cycle can be described as a combination of almost simultaneous processes of proliferation and apoptosis. During apoptosis, the cell reduces its volume, losing contact with neighboring cells. During necrosis, cell swelling occurs and plasma membrane cohesion is lost. Using the cell impedance measurement method, the changes that occur can be detected because it allows indirect cell counting and detection of cell membrane cohesion. These processes change the morphology of the cells covering the electrode, enabling their quantitative detection.

**Fig.1:**
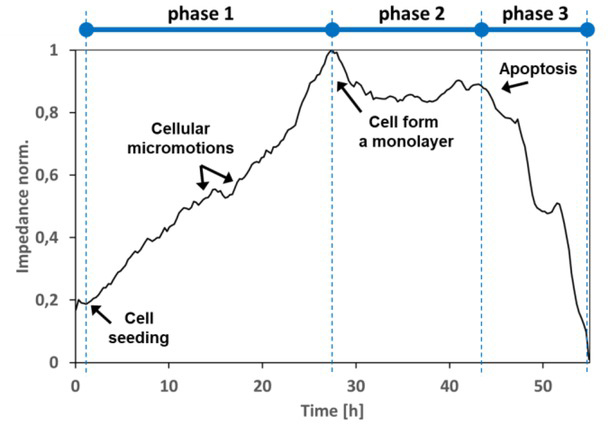
Impedance response (normalized) of mouse fibroblast cells on measured by an ECIS sensor array with a golden electrode at 16 kHz, showing various cellular morphological changes.


The main component of the measurement system is a pair of small gold electrodes deposited on a test substrate at the bottom of a cell culture dish. Often, there is an insulating polymer on the metal layer that limits the contact area of the electrode with the culture, which allows monitoring of changes in the measured parameters for a smaller number of cells. The attachment and spread of cells on the electrodes affect the flow of current, which changes the measured impedance value. Impedance changes are important dynamic information about cell shape and movement. The primary process of increasing the number of cells through reproduction and division is proliferation. It causes an increase in the coverage of the electrodes by the non-conductive cell membrane. This results in a significant increase in the measured impedance value. Monitoring the cell proliferation process using impedance measurement is not a direct method of counting the number of cells, but it is a recording of changes in the measured values resulting from changes in the coverage of the measuring electrode area. Thus, there is a close relationship between the number of cells and the normalized impedance value (Fig. 1). In this paper, the min-max normalization was used, represented by the formula: [14]:

$$x^{\prime }=\frac{x-x_{\min}}{x_{\max}-x_{\min}},$$

where *x'* is the normalized value within the range from 0 to 1, while *x*, *x_min_*, *x_max_* mean, respectively: the measured value and the smallest and largest value of the normalized interval.

For simplicity, the circuit can be represented as a parallel connection between a resistor and a capacitor. The measurement is carried out by switching on an AC current, usually less than 1 mA, that flows through the measurement system. Resistance (R) and reactance (X) measurements are made, allowing the calculation of impedance modulus |Z|, capacitance (C), and phase angle (φ). At frequencies below 2 kHz, a significant amount of current flows through the intercellular spaces, providing information about cell adhesion. The use of a relatively high frequency (~40 kHz) causes current flow directly through the cell membrane. The reading of each parameter is plotted as a point versus time: in ohms for resistance and impedance or farads for capacitance. As a result, information about the amount of electrode coverage by cells is obtained. By performing numerous experiments, the influence of the materials used on the tested biological system is described on the basis of such behaviors as adhesion, proliferation, or migration of cells. Depending on the type of process being monitored, the information obtained makes it possible to identify interactions between the tested material and the living organism under specific conditions [15, 16].

The first observed phenomenon that can provide information about the biocompatibility of a given material is cell adhesion. In biological terms, it is the permanent and irreversible fusion of a cell with the surface of a solid. Adhesion significantly influences cell migration and growth. The adhesion and immobilization of cells, caused by their metabolic changes and environmental conditions, is related to a specific type of adhesion, called adherence [17].

Another observable process is cell migration which is defined by movement speed and directional constancy. Cell migration is particularly important in research into the ability of tissues to regenerate in the presence of implanted material. This process depends mainly on the type of surface with which the cells are in contact. In the case of the absence of external stimuli, cellular activity is random and their movement is chaotic. It is important to mention that cell spreading is strongly correlated with wettability. This term refers to the freedom with which a substance is spread over the surface of a material. The above phenomena, related to cell adhesion and spreading ability, affect the most important determinant of biocompatibility, which is proliferation. In a general sense, it is the ability of cells to reproduce, which is determined by the ratio of factors influencing their growth to toxic or other factors slowing this process. During quantifying growth rates, it is important to measure the number of living cells adhering to a given surface. At the testing time, the number of cells exposed to a test substance is compared to the number of cells grown on a control material, which is usually a material commonly considered to be biocompatible. Based on described phenomena, it is possible to determine the viability of the study population.

The analysis is performed by observing the cell life cycle, which is composed of several phases (Fig. 1). The duration of the experiment depends on the user and can take from a few seconds to several days. As cells grow and cover the electrodes, the current is inhibited in a manner related to the number of cells covering the electrodes, the morphology of the cells, and the nature of cell adhesion (resistance increases - this is phase 1). Based on preliminary observations on adhesion, the effect of surfaces on cellular dysfunction can be tentatively determined. When cells are intolerant to the substrate material, poor adhesion with the surface is observed. While cells are in contact with the material, the cells begin to multiply and migrate across the surface of the substrate. When the culture covers the entire surface of the culture dish, the process of cell proliferation and spreading stops.

Phase 2 represents stabilization of the cell culture, where stabilization of the electrical parameters is also evident. In phase 3, the cells die and adhesion decreases, thus decreasing resistance and increasing capacitance. This stage relates to cell death, and more specifically to Programmed Cell Death (PCD). In the natural environment, this process is related to the development of organs and is aimed at the elimination of damaged or infected cells. This provides a state of balance between the formation of new cells and the destruction of old ones. On microscopic observation, it is evident in the detachment of cells from the bottom of the culture dish.

### Electrode design and fabrication

Following the example of commercial plates of the 8W10E type, a mask with eight pairs of 200 μm × 200 μm comb electrodes (single finger width × finger spacing) on a single substrate was designed. With an identical pin arrangement to the standard plates supplied by the measurement equipment manufacturer, the fabricated set will be able to adapt for conducting experiments in the ECIS® Z-Theta system apparatus. Above each electrode pair, there is a special container attached with biocompatible silicone. The cell cultures under investigation are applied to its interior. To provide the necessary nutrients for culture, the test cells are surrounded by a medium containing the appropriate nutrient.

**Fig. 2:**
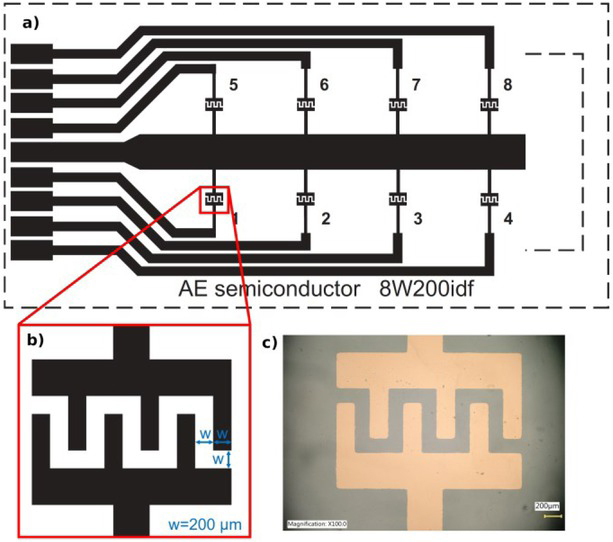
The technological mask design of the cell impedance measurement test substrate, compatible with the base station of a commercial measurement device (a) view of the whole substrate, (b) single pair of electrodes, (c) photo of the copper electrodes taken with a Keyence VHX 5000 optical microscope.

Looking for an alternative to the commercially used but expensive gold, which is applied in commercial matrices, it was decided to test titanium (Ti), nichrome (NiCr), and copper (Cu). The selection was dictated by the different degrees of biocompatibility of these materials [18, 19] and their wide application in Lab-On-a-Chip devices. Processes known from microelectronic technologies were used to fabricate the structures. A thin layer of metallization was made on a polycarbonate substrate using the magnetron sputtering method. The obtained layers were as follows: Cu 100 nm, Ti 400 nm, NiCr 200 nm, Ni 200 nm. Then, the process of photolithography and selective etching of the metal were performed [20, 21].

### Tissue culture

For the experiment, mouse fibroblast cells - clone NCTC 929 (L cell, L-929, L-strain derivative) were used, which were grown according to the instructions in a complete EMEM culture medium, supplemented with 10% heat-inactivated fetal bovine serum and antibiotics (100 IU/ml penicillin, 10 mg/ml streptomycin, 25 μg/ml amphotericin B) in the Galaxy 170R incubator, under controlled growth conditions, constant humidity and constant air saturation of 5% CO_2_. After the proliferation and stabilization of the cells (about 7– 14 days), when the culture reached at least 75% confluence, the next stage of the research was started. It consisted in placing the matrices in the holders of the measuring station and then placing them in an incubator at 37°C and 5 % CO_2_ concentration. Before the cells were inoculated, the test plates containing the electrodes were incubated for 24 hours with only a medium. After the conditions stabilized, the measuring matrix was taken out and a mixture of 540 μl of fresh medium and 60 μl of cell suspension was applied inside it. The seeding in the matrices was carried out at 600 μl of suspension per each of 8 wells (~1.2 · 10^5^ cells/ml). The termination of each culture was dependent on the obtained results, which were monitored during the experiment.

### Ethical approval

The conducted research is not related to either human or animal use.

## Results and discussion

One of the main goals of the experiment was to achieve sufficient biocompatibility for some applications. Cell culture was expected to develop properly on the produced matrices and the obtained measurement results would allow the analysis of cell activity. At the same time, due to the presence of materials with low biocompatibility, the dispersion of the measured parameters between the wells and their different character compared to the case of using substrates with gold electrodes, will still be acceptable. The criterion to be met by the substrate material was to show a degree of biocompatibility that would allow the cells to be cultured. The least desirable situation would be such a strong negative effect of the materials that would cause the cells to die immediately after they were injected into the wells and thus prevent any evaluation of the experiment.

**Fig. 3:**
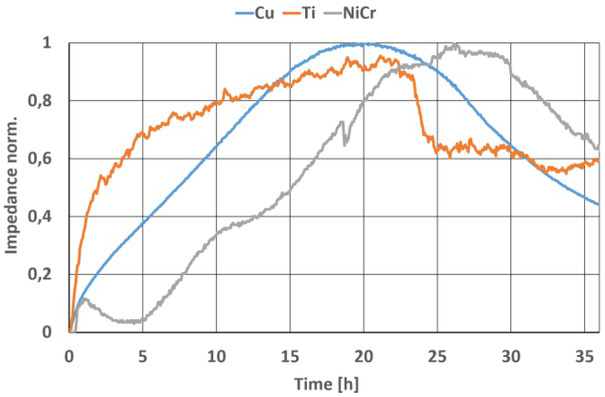
Impedance response measured by an ECIS sensor array with Ti, NiCr, and Cu electrodes at 16 kHz.

After cell culturing on the selected materials, the collected results were normalized. In this form, changes in measured values can be observed and the nature of these changes can be compared without the influence of the resistance of the metallization paths. Cultures performed in all prepared test substrates showed the expected nature of changes in the measured values. Cell proliferation and migration occurred in nearly all of the culture wells. After 30 hours of the experiment, a steady decrease in impedance values was observed in each of them, resulting from cell apoptosis (the last phase of cell culture). For each of the tested materials, a representative well was selected and the impedance values measured for them are presented in Figure 3 for a signal with a frequency of 16 kHz. The changes seen throughout the measurements indicate that a complete cell life cycle has occurred.

Phase 1, in which cells attach to the substrate, proliferate, and migrate most rapidly occurred for cells cultured in the presence of biocompatible titanium. Phase 2 lasted differently for each material e.g. for toxic copper it was unstable. Immediately after the entire bottom surface of the well was filled, there was a smooth transition to phase 3. Small changes in impedance values are also seen, indicating negligible cell migration. Compared to cultures in the presence of reactive NiCr, where the values have a larger fluctuation than in the presence of Cu in both phase 1 and phase 2. Nevertheless, the trend of changes is as expected for both materials. The largest cell movements can be observed for cultures with titanium, which as expected should have the least effect on cell activity.

**Fig. 4:**
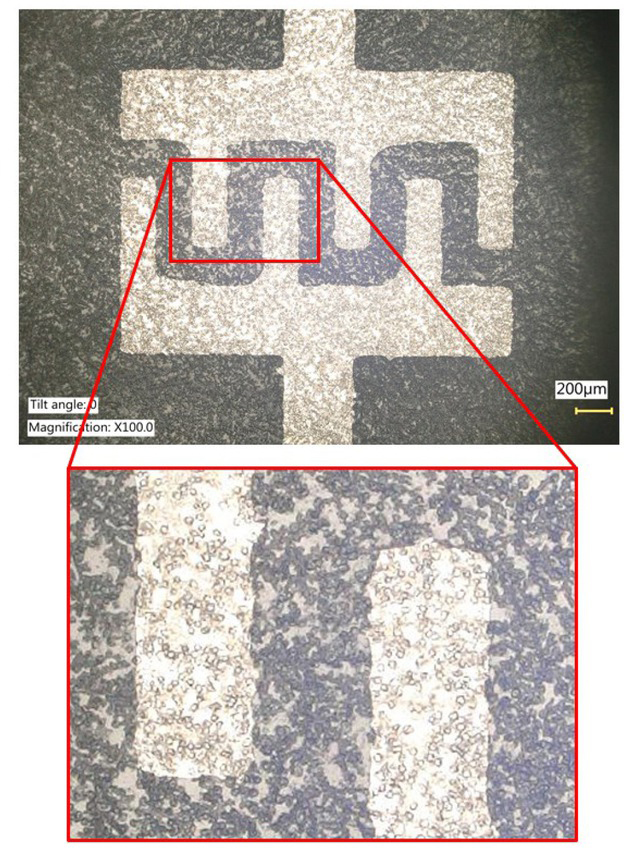
Nichrome capacitor electrode with the dead cells after the experiment (magnification ×100).

Considering all the observed changes, it can be concluded that the use of fabricated matrices with Ti, NiCr or Cu electrodes allows short-term biological studies. The cells in the wells were not killed as a result of the negative influence of the material at the beginning of the experiment. The culture was successful and the measurement data obtained allowed us to observe changes due to cellular activity (Fig. 4). All test substrates used provided results indicating potential use in other biological studies.

## Conclusion

Three metals, characterized by different parameters and varying degrees of biocompatibility, were selected for the experimental experiment. Mouse fibroblast cultures were fully cycled in the presence of selected materials. For each of them, there was a different character of changes in the measured impedance values due to the different degrees of reactivity of a given material with the surrounding tissues.

The criterion to be met by the substrate material was to exhibit a degree of biocompatibility that would allow cell culture to take place. The experiment showed that it is possible to conduct such studies on the biocompatibility of various materials compared to the activity of cells grown on gold electrodes.
